# Towards a typology of social protection for migrants and refugees in Latin America during the COVID-19 pandemic

**DOI:** 10.1186/s40878-021-00265-x

**Published:** 2021-11-16

**Authors:** Marcia Vera Espinoza, Victoria Prieto Rosas, Gisela P. Zapata, Luciana Gandini, Alethia Fernández de la Reguera, Gioconda Herrera, Stephanie López Villamil, Cristina María Zamora Gómez, Cécile Blouin, Camila Montiel, Gabriela Cabezas Gálvez, Irene Palla

**Affiliations:** 1 School of Geography, Queen Mary University of London, Mile End Rd., London, E1 4NS UK; 2 Programa de Población, Facultad de Ciencias Sociales, Universidad de la República, Constituyente 1502, CP 11100 Montevideo, Uruguay; 3 Centro de Desenvolvimento e Planejamento Regional (CEDEPLAR), Universidade Federal de Minas Gerais (UFMG), Av. Antônio Carlos, 6627, Belo Horizonte, MG 31270-901 Brazil; 4 Instituto de Investigaciones Jurídicas, Universidad Nacional Autónoma de México, Circuito, Mario de La Cueva s/n, C.U., Coyoacán, 04510 Mexico City, CDMX Mexico; 5 Departamento de Sociología y Estudios de Género, Facultad Latinoamericana de Ciencias Sociales, FLACSO Ecuador, Calle La Pradera E7-174 y Av. Diego de Almagro, Quito, Ecuador; 6 Facultad de Derecho, Ciencias Políticas y Sociales, Universidad Nacional de Colombia, Ciudad Universitaria, Bogotá, Colombia; 7 Dpto. Derecho Internacional Público y Relaciones Internacionales, Facultad de Derecho, Universidad de Sevilla, Avda. La Enramadilla, 18-20, CP 41018 Seville, Spain; 8 Pontificia Universidad Católica del Perú (PUCP), Avenida Universitaria 1801, San Miguel, Lima, Peru; 9 Instituto de Democracia y Derechos Humanos, Pontificia Universidad Católica del Perú (IDEHPUCP), Calle Tomás Ramsey 925, Magdalena, Lima, Peru

**Keywords:** Latin America, Covid-19, Social protection, Migrants, Refugees, Migration governance, Inclusion, Exclusion, Migration policy

## Abstract

The COVID-19 health crisis has put to the test Latin America’s already precarious social protection systems. This paper comparatively examines what type of social protection has been provided, by whom, and to what extent migrant and refugee populations have been included in these programmes in seven countries of the region during the COVID-19 pandemic, between March and December 2020. We develop a typology of models of social protection highlighting the assemblages of actors, different modes of protection and the emerging migrants’ subjectification in Brazil, Chile, Colombia, Ecuador, Mexico, Peru, and Uruguay in relation to Non-Contributory Social Transfer (NCST) programmes and other actions undertaken by state and non-state actors. The analysis is based on 85 semi-structured interviews with representatives of national and local governments, International Organisations, Civil Society Organisations, and migrant-led organisations across 16 cities, and a systematic review of regulatory frameworks in the country-case studies. The proposed typology shows broad heterogeneity and complexity regarding different degrees of inclusion of migrant and refugee populations, particularly in pre-existing and new NCST programmes. These actions are furthering notions of migrant protection that are contingent and crisis-driven, imposing temporal limitations that often selectively exclude migrants based on legal status. It also brings to the fore the path-dependent nature of policies and practices of exclusion/inclusion in the region, which impact on migrants’ effective access to social and economic rights, while shaping the broader dynamics of migration governance in the region.

## Introduction

The COVID-19 sanitary crisis has put to the test Latin America’s already precarious social protection systems. The pandemic also hit the region in the midst of one of the largest human displacements in its recent history, with the number of people displaced across borders growing by 400% in the last decade (UNHCR, 2020). States responses to this increased mobility have been characterised by the adoption of multiple *ad-hoc* and temporary measures to manage migration (Acosta et al., 2019; Gandini et al., 2019, 2020b), producing migrant irregularity (Thayer, 2019) and triggering increasing levels of xenophobia across the region (Freier & Vera Espinoza, 2021). This *ad-hoc* approach to migration governance has been accompanied by a myriad of social protection actions on the part of the state, International Organisations (IOs) and Civil Society Organisations (CSOs) intended to cope with the emergency, leaving little room for the long-term inclusion of migrants beyond an epistemology of exceptionalism (Menjivar et al., 2019; Mountz, 2020).

It has already been well documented how the pandemic has exacerbated the pre-existing vulnerabilities of migrant and refugee populations in the region, given their high rates of job informality, overcrowded and precarious living conditions, and in some cases, limited access to health services and social protection (Bengochea et al., 2021; The Lancet, 2020; Zapata & Prieto, 2020). Relevant knowledge has also been developed in relation to changes in social protection systems and safety nets in Latin America during COVID-19 (Blofield et al., 2020; Williams & Martinez, 2020). Building on this work, this paper provides a comparative and in-depth analysis of social protection actions in Brazil, Chile, Colombia, Ecuador, Mexico, Peru, and Uruguay with regards to three key issues: (1) what type of assistance is provided, (2) who provides the assistance, and (3) to what extent migrant and refugee populations^1^ are included in these responses. We look at the inclusion (or lack thereof) of migrant and refugee populations in the Non-Contributory Social Transfer (NCST) programmes and other actions undertaken by governments, IOs and CSOs between March and December 2020, and what this tells us about the place of migrants’ social rights and citizenship in the eyes of the state. We draw on 85 online semi-structured interviews conducted with representatives of the above-mentioned stakeholders, combined with a systematic review of legal frameworks—constitutions, laws, and decrees—in the seven country-case studies.

We develop a typology of models of social protection in the context of COVID-19 to show the assemblages of actors providing social assistance, the modes of protection and the emerging migrants’ subjectification in the seven countries analysed. We argue that there are different levels of inclusion of migrant and refugee populations in pre-existing and new NCST programmes across the region that are furthering notions of protection which are contingent and crisis-driven, imposing temporal limitations that often selectively exclude migrants based on legal status. This typology brings to the fore the path-dependent nature of policies and practices of exclusion/inclusion in the region, which impact on migrants’ effective access to social and economic rights, while shaping the broader dynamics of migration governance in the region.

This article is organised in six parts. In the first section, we draw on critical studies on how notions such as humanitarianism, exceptionalism, and crisis may undermine migrants’ rights and citizenship in relation to social protection. In the second section, we outline the methodological approach and the limitations of the study. In the third section, we provide an overview of the legal, social and economic rights afforded to migrants and refugees in the seven countries before COVID-19. In the fourth section, we turn to the analysis of pandemic NCST initiatives in each country and the extent to which their design and implementation includes or excludes migrants and refugees. On these bases, we then propose a typology of social protection in relation to the assemblage of actors, modes of protection and the emerging subjectification of migrants during the pandemic. We conclude by highlighting the implications of these findings for the governance of mobility in Latin America during, and beyond, the pandemic.

## Theoretical framework

### Social protection and migrants’ social rights

Social protection emerged in the 1990s as a policy framework employed to address poverty and vulnerability in Latin America as a result of economic crises and the shift towards structural adjustment policies (Barrientos, 2010). Almost all Latin American states have since designed different kinds of social protection strategies, mainly geared towards poverty reduction. Barrientos (2010) identifies three types of policy strategies depending on whether they focus on mitigating risks, attending needs, or are related to entitlements and rights. The United Nations Human Rights Council emphasises that “human rights obligations relate not only to the final outcome of social protection programmes, which is to ensure the enjoyment of at least minimum essential levels of economic, social and cultural rights, but also to the process through which such programmes are designed and implemented” (Sepulveda & Nyst, 2012: 11).

This has several implications for our understanding of social protection policies in the context of migration. First, the existence or absence of formal rights for migrants in the reception countries’ legal frameworks is an important factor in determining the scope of social protection actions. Second, it may also indicate states’ obligations to implement long-term and sustainable programmes, as opposed to exceptional or emergency actions. And third, the existence of formal rights could empower migrants to claim them.

At the same time, the spectrum exclusion/inclusion is fundamental to debates on citizenship and migration, as it raises the question on the extent to which migrants should be granted rights. De Lucas (2002) argues that social rights, such as the right to work and to health, are a *sine qua non* condition for migrants’ integration, which can be broadly understood as “the process of becoming an accepted part of society” (Penninx & Garcés-Mascareñas, 2016: 14). A first issue with social rights is that this category of rights has been understood as social benefits rather than true rights with real justiciability (De Lucas, 2002). A second issue is the use of nationality or immigration status criteria to differentiate and stratify people, generating unequal conditions and uneven access to social rights (Asa & Ceriani, 2010).

In this context, it is common for states to negotiate to what extent social rights are guaranteed to migrants and refugees (De Lucas, 2002), usually based on bureaucratic labels such as irregular migrants, refugees, asylum seekers or humanitarian migrants with complementary protection status. The proliferation of new legal categories to face the challenges of increasingly complex mixed migration flows has created fragmentation (Crawley & Skleparis, 2018; Zetter, 2007) and has had consequences for access to rights (Morris, 2016). In light of this, Asa and Ceriani (2010) emphasise that it is urgent to question the concepts of citizenship and national sovereignty at the centre of this process of differentiation of rights, which differ from the universality, progressiveness and dynamism of human rights.

The definitions proposed by Davidson and Castles (2000) of *quasi-citizens*, *denizens* and *margizens* are relevant to our analysis, as they help to explain the extent to which migrants and refugees are included or excluded from COVID-19 governmental responses. *Denizens* or *quasi-citizens* are foreigners with a legal and permanent resident status, while *margizens* include a vast group of persons in the margins such as undocumented migrants, asylum-seekers, or legal citizens who have lost their status, among others. Migrants and refugees with temporary permits would qualify as *margizens* as they face numerous barriers for their integration and are denied many rights as non-citizens. Yet, depending on the country, *margizens* may not be completely deprived of rights.

Here, Morris’ (2016) concept of civic stratification is useful to understand how social protection policies may contribute to deepening social inequalities, given that even if migrants and refugees are legally entitled to certain rights, unequal access to social protection may occur. Thus, rather than a dichotomy between inclusion and exclusion, there may be a continuum of social policies that place migrants and refugees in different positions in the social stratification ladder. This stratification is linked to notions of (non)citizenship socially attributed to migrants, considering that crossing borders supposes the demarcation of different rights (Balibar, 2002).

Another debate that frames our analysis is the extent to which social protection actions respond to integral and stable policy frameworks or to discreet emergency actions framed in a humanitarian narrative. Usually, humanitarian action is associated with the work of international and non-profit organisations, yet states are increasingly using the language of humanitarianism in the implementation of policies toward migrants and refugees, replacing a politics of rights and justice with an ethics of suffering and compassion (Fassin, 2012). In doing so, humanitarianism tends to render the passive and suffering body, instead of the deserving citizen, as a sort of proof to justify state action and intervention (Ticktin, 2011). In this vein, Dijstelbloem and van der Veer (2019) discuss the apparent contradictions of humanitarian border dynamics, characterised by the articulation of both care and control practices by multiple actors in response to growing mixed migration flows (Walters, 2011). These simultaneous practices intensify the vulnerability of migrant and refugee populations in contexts where social protection mechanisms are limited. Yet, despite the multiplicity of actors in these humanitarian contexts and the increasing privatisation of social assistance, it is the state that ultimately has the sovereign power of granting social and economic rights (Jones et al., 2017).

While these trends have been widely discussed in the European context (Ticktin, 2011), they are more recent in Latin America. The academic debates in the region have addressed the way in which some policies, while clothed in moral universals and humanitarian imperatives, are ultimately aimed at the management, control, and exclusion of mobile populations (Finn & Umpierrez de Reguero, 2020; Herrera & Berg, 2019; Ramirez, 2020; Stang et al., 2020). Indeed, with the advent of the recent Venezuelan and Central American exodus, the idea of a humanitarian crisis emerged across the region, with several studies and some international organisations claiming that the recent massive migration has been a response to numerous human rights violations and severely deteriorated socioeconomic conditions threatening survival (Freier, 2018). Against this background, terms such as “migration in the context of crisis” emerged to explain the drivers of this migration flow, alongside “migration crisis” discourses to justify the exceptionalist nature of the political-institutional responses adopted by many Latin American receiving countries (Freitez, 2019; Gandini et al., 2019; Zapata & Tapia, 2021). However, as some migration scholars have argued, the idea of a migration crisis could become a magnifying lens to decipher existing trends towards a politics of humanitarianism (Cantat et al., 2019), as well as a ‘productive’ word that the states can use to justify their actions (Mountz, 2020). This is because the crisis narrative that we see taking a hold across the region is key to rendering the responses to mobility as something ‘exceptional’, justifying humanitarian discourses and practices (Menjivar et al., 2019; Stang et al., 2020). In this scenario, the growing role of international organisations such as the International Organisation for Migration (IOM) and the United Nations High Commissioner for Refugees (UNHCR) in the regional response to these crises is closely related to the idea of migration management, under the slogan of safe, orderly and regular migration, aimed to deal with states’ sensibilities towards interference with their sovereignty (Pécoud, 2018).

In the next sections, we look at how this humanitarian crisis discourse has contributed to disengaging some states from adopting inclusive social protection policies towards migrants and refugees, relying instead on exceptional policies mainly geared by international organisations and other non-state actors, and how these narratives have contributed to particular modes of protection and the subjectification of migrants.

## Data and methodological approach

Our analysis is based on a comparative assessment of seven country-case studies in Latin America: Brazil, Chile, Colombia, Ecuador, Mexico, Peru, and Uruguay. The case selection responded to practical reasons related to the authors’ expertise and/or location during the COVID-19 outbreak, as well as methodological considerations. In particular, these countries cover a diverse range of assemblages regarding migration policy and social protection, as well as political-institutional responses to mitigate the socio-economic impacts of the pandemic. However, they also have some commonalities such as: (1) structural inequalities, (2) having experienced recent transformations in their migration dynamics, with some suddenly turning from sending to transit and destination countries, and (3) undergoing changes in their migration legal framework.

The study employs a qualitative methodology combining a systematic analysis of legal frameworks and online interviews with key stakeholders. On the one hand, we reviewed over forty legal instruments that rule migration in the country-case studies including constitutions, laws, decrees, and administrative acts to examine (1) whether migrants and refugees were explicitly mentioned as subjects of civil rights deserving equal treatment on par with nationals, (2) the circumstances under which social and economic rights were granted to them, and (3) the main existing and new NCST programmes introduced during the pandemic.

On the other hand, based on a purposive sampling, we conducted 85 online semi-structured interviews^2^ with three distinct groups of actors that directly or indirectly assist migrant and refugee populations in each country: representatives from national and local governments, International Organisations (IOs), and Civil Society Organisations (CSOs). IOs included UN agencies, primarily, IOM and UNHCR, while CSOs included local, national and international non-governmental organisations (NGOs), religious and non-religious, as well as migrant-led organisations. Aware of the fact that in the federative states, local governments play a crucial role in the provision of some social and economic services, apart from seven major cities in the country-case studies, the fieldwork also included at least one border city in each country, for a total of 16 urban contexts analysed. Details on the number of interviews conducted and type of actors by country are included in the Appendix (Table 4).^3^

The interview schedule was organised in four sections and included questions related to (1) changes in migration and refugee policy and practice during the pandemic, (2) the conditions and access of migrants to health, housing, labour, and social protection during the pandemic, (3) attitudes towards migrant and refugee populations, and (4) changes in the modes of working and/or type of assistance provided by the different actors interviewed. Despite minor variations to address the specificities of each type of actor, we used the same interview schedule across all seven countries. Given the mobility restrictions imposed by COVID-19, the interviews were conducted online by audio/video conferencing, between June and September 2020. Interviews lasted between 40 min and 1 h, and informed consent was given by all participants. The study received approval by the ethics committees of two of the authors’ higher education institutions.^4^

The interviews were transcribed and coded into themes set in advance by the research team in a group discussion. The data was then inputted in a common systematization matrix, which enabled triangulation of responses by type of actor across the case studies. The narratives collected in the interviews are imbued throughout the analysis and interviewees have been cited in the paper where appropriate.

This study is not devoid of limitations. We identified an absence of national data disaggregated by migration status, nationality or place of birth, to gauge to what extent migrant and refugee populations effectively accessed NCST schemes before and during the pandemic. In fact, during the interviewing process we tried, unsuccessfully, to collect precise data from the informants on the number of applications and benefits granted broken down by any migration identifier. Even harder was to obtain this data for all seven countries disaggregated by age, sex, race and ethnicity, which highlights the importance of pursuing the type of qualitative analysis conducted in this study. This qualitative analysis, in turn, allows us to explore the complexity and nuances of processes, norms, and implementation of programmes by type of actor in each country. Future studies should expand the geographical and demographic scope of our analysis while attempting to integrate an intersectionality approach, where possible.

## Normative context: migrants’ social and economic rights before COVID-19

The migration dynamics of most Latin American countries have undergone substantial transformations over the past 20 years. Traditionally an emigration region, Latin America is increasingly becoming a transit and destination area, mainly as a result of the tightening of migration policies and the deterioration of employment markets in traditional destinations (Gandini et al., 2020b). In parallel with this reconfiguration of flows, changes have been made to the migration frameworks of virtually all countries, to include a human rights perspective and the Cartagena refugee protection framework (Acosta, 2018; Jubilut et al., 2021).

Regularisation procedures and access to rights are shared paradigms in the legal frameworks of countries such as Brazil, Chile, Colombia, Ecuador, Peru and Uruguay (CELS and CAREF, 2020). However, variations appear when looking at migrants and refugees’ full guarantee of social and economic rights, ranging from those who recognise them explicitly and comprehensively, to others with unspecified rights, or rights conditioned to having a regular immigration status. In Table 1, we propose a classification of legal frameworks on the basis of two criteria: clarity or ambiguity of legal language -if the aforementioned Constitution, laws, and decrees clearly and specifically enumerate immigrants’ social rights- and whether guarantee of social and economic rights (health, education, work, food and housing) is conditioned to immigration status.

**Table 1:** Migration Legal Frameworks in selected Latin American countries

Country	Equality before the law			Extension of social and economic rights	Legal framework inclusion of specific social rights
	Implicit	Explicit	Includes irregular migrants		
Brazil		☑ Federal Constitution [1988] (Art. 5)	☑ Included	Health, education, labour, social assistance and security (Migration Law, Art. 4)	Legal clarity and full inclusion
		Migration Law 13,445/2017 (Art. 3, XI) & Refugee Act 9,474/1997. (Art. 5, II)			
Colombia		☑ Political Constitution (Art. 100)	☒ Not included, except for emergency health and education	Civil rights (Political Constitution, Art. 100)	Legal ambiguity and partial inclusion
		Law 1465/2011: National Migration System. CONPES 9650/2018. Decree 2840/2013 (refugees)			
Chile	☑ Political Constitution [1980] (Art. 19)	☑ Refugee Law 20,430/2010 (Art. 13)	☑ Included for education, health, and labour rights (Oficio ordinario no. 07/1008 (1531); Supreme Decree no.67)	Civil rights (Political Constitution, Art 19); Health, education, labour, social protection (Refugee Law, Art. 13)	Legal ambiguity and partial inclusion
Ecuador		☑ Political Constitution [2008] (Art. 9, 34, 35, 40, 41). Expanded Refugee Registry (2009)	☑ Included	Health, education, labour, social protection (Political Constitution, Art. 3, 34, 35, 40)	Legal ambiguity and full inclusion
		Organic Law of Human Mobility (Refugee included) (2017)			
		National Human Mobility Plan (2018)			
		Decree 826. Exception Visa for Humanitarian Reasons (VERHU) (2019)			
Mexico	☑ Political Constitution [1917] (Art. 1)	☑ Migration Law (Art. 8 and 15)	☑ Included for health and education services	Health and education for all migrants (Migration Law, Art. 8)	Legal ambiguity and partial inclusion
		☑ Refugee, Complementary Protection and Political Asylum Law (Art. 44)		Health, education, labour & identity for refugees and regular immigrants (Migration Law, Art. 15 & Refugee Complementary Protection and Political Asylum Law, Art. 44)	
Peru	☑ Political Constitution (Art. 2)	☑ Migration law and regulations: Legislative Decree 1350, Supreme Decree 007–2017-IN	☑ Included for access to justice, health, education and labour	Health, education and labour (Legislative Decree no. 1350, Art. 9; Migration Law & Refugee Act, Art. 14)	Legal ambiguity and partial inclusion
		☑ Refugee Act: Law 27,891, Supreme Decree 119–2003-RE			
Uruguay	☑ Constitution of the Republic [1967] (Art. 7 and 8)	☑ Migration Law (18,250/2008) (Art. 7)	☑ Included for education and health services and labour	Health, housing, education, labour and social protection (Migration Law, Art. 8 & Law on the Right to Refuge and Refugees, Art. 20)	Legal clarity and full inclusion
		☑ Law on the Right to Refuge and Refugees (18,076/2006) (Art. 20)			

Source: Own elaboration based on review of Legal Regulatory Frameworks in each of the seven country case studies

Following these criteria, we identify three groups of countries. The first group, *legal clarity and full inclusion,* is made up of Uruguay and Brazil, which are characterised by a clear legal framework where migrants and refugees’ social rights are fully recognised regardless of migration status and equality before the law is guaranteed. Additionally, these two countries stand out in the regional context for their robust social protection systems, based on an array of contributory and non-contributory social transfers, with the latter targeting households composed of the elderly, children and adolescents (Blofield et al., 2020). In practice, Uruguay requires the possession of an identity card while in Brazil, the ID required to access social protection programmes is not conditioned by legal status, facilitating effective universal access to NCSTs.

The second group, *legal ambiguity and full inclusion*, is composed only of Ecuador, whose laws, in spite of a certain ambiguity, guarantee the full inclusion of immigrants and refugees and equality of rights between foreigners and Ecuadorian nationals (Table 1). The 2008 Political Constitution and the 2017 Organic Law of Human Mobility recognise the social and economic rights of migrants and refugees regardless of immigration status. However, subsequent decrees introduced some ambiguity to Ecuador’s legal framework. For instance, Decree 804 (June 2019) excludes non-Ecuadorian nationals from accessing existing cash transfer programmes.

Finally, the third group*, legal ambiguity and partial inclusion* is composed of Chile, Colombia, Mexico and Peru. These four countries present ambiguity with regards to the rights guaranteed for both regular and irregular migrants, which results in several obstacles to guarantee full and effective inclusion. Constitutions in these countries establish equal civil rights for all individuals. However, in countries with migration and/or refugee laws some of the basic social rights are limited to regular migrants.

In the case of Chile, several articles of the Political Constitution are implicitly applicable to migrants, and even though irregular migrants are not included in this legal framework, the country has adopted specific decrees and regulations aiming to guarantee access to education and health, independently of residency status, as well as equal labour rights. In Peru for example, the Refugee Law, inter alia, guarantees labour rights for asylum seekers, and the Migration Law guarantees the right to health, education, and work for all migrants, including those with irregular status. At the same time, the law states that access to rights depends on specific norms issued by different Ministries, which undoubtedly constitutes a huge barrier to migrants’ effective access. For its part, Colombia has given the first steps to resolve institutional gaps regarding migrants’ rights through a multiplicity of temporary measures.^5^ For instance, access to emergency health services and education is available to irregular Venezuelan migrants, while migrants with a *Permiso Especial de Permanencia* (PEP) [Special Stay Permit] have been granted access to health, education, and the labour market. In contrast, Mexican Migration and Refugee law guarantee access to educational and emergency health services to all migrants regardless of their status but limit economic and social inclusion to those with regular status, and allow family reunification only for refugees. In the cases of Colombia, Peru and Mexico, contingent circumstances such as the great inflows from, respectively, Venezuela in 2016 and the 2018 Central American Caravans, led to the creation of instruments to regularise (temporarily in some cases) immigrant populations in order to facilitate access to health, education and labour programmes (Gandini et al., 2019, 2020a).

## Social protection in the context of the pandemic: pre-existing and new programmes

Table 2 systematises the narratives by the three types of actors interviewed in the seven country case studies, with regards to programmes and actions to mitigate the social and economic effects of the pandemic among the migrant and refugee populations. We focus on NCST programmes and actions undertaken by governments, IOs and CSOs, including cash and in-kind benefits such as food vouchers, food baskets, and provision or guarantee of goods and/or services. Government programmes are limited to those that were maintained, expanded, or created during the pandemic. These include both *Conditional Cash Transfer* (CCT) programmes—such as *Bolsa Família (PBF)* [Family Grant] in Brazil and *Asignaciones Familiares (AFAM)* [Family Allowances] in Uruguay—and non-conditional new emergency transfers created to respond to the pandemic.

**Table 2:** Non-contributory social transfers (NCST) during the pandemic in selected Latin American countries by type of provider and inclusion/exclusion of migrant and refugee populations

Country	NCST from national government					NCST from International Organisations (IOs) and Civil Society Organisations (CSOs)			
	Inclusion of migrants/refugees					Inclusion of migrants/refugees in IOs programmes		Inclusion of migrants/refugees in CSOs initiatives	
	Main programmes	Status	Requirements for inclusion	Effective inclusion	Reason for exclusion	Main programmes	Criteria for inclusion	Main initiatives	Criteria for inclusion
Brazil	*Bolsa Família* (PBF)	Increase in existing transfer and expansion to new recipients	PBF: Regular migration status [CPF or Valid ID for registration in social programmes registry (CadÚnico). CPF is not conditioned to regular migration status]	PBF: Full inclusion	PBF: Over income threshold	Cash transfers Direct food provision NGO financing of food provision Hygiene kits [personal and household]	Open to anyone in need (nationals and non-nationals) Most vulnerable individuals/families are prioritised Some funds are earmarked for humanitarian response for Venezuelans	Cash transfers Direct food provision Personal and household hygiene kits	Open to anyone in need (nationals and non-nationals) Most vulnerable individuals/families are prioritised for cash transfers
	*Auxílio Emergencial* (AE)	New transfer	Regular migration status [CPF and valid ID. expiration dates of government-issued IDs were extended]	Full inclusion [Despite initial implementation problems most migrants and refugees were contemplated]	Digital registration opened to all In-person cashing-in of benefit requires possession of valid migration or refugee document				
Chile	*Subsidio Único Familiar (SUF)*	Maintenance	Regular migration status [National ID with ‘Unique National Number’ RUT. A valid RUT is conditioned to regular migration status]	Limited to regular migrants	Irregular migration status	Financing for NGOs, municipalities and national government Cash transfers Vouchers and direct food provision Financing of shelters and lodging	Open to migrants, asylum seekers and refugees Most vulnerable groups are prioritised Venezuelans and vulnerable migration groups are prioritised	Cash transfers Direct food provision Hygiene kits	Open to migrants, asylum seekers and refugees
	Subsidies^1^	New transfers	Regular migration status [Valid national ID with RUT and previous registration in other NCST programmes—household social registry]	Limited to regular migrants and population [discretionality]	Irregular migration status and no previous enrolment in NCST pre existing programmes				
Colombia	*Familias en Acción* (FA)	Maintenance	Colombian nationality	Exclusion	Migrants and refugees are not eligible	Cash transfers Direct food provision Hygiene kits -Financing for NGOs, municipalities and national government	Open to migrants, asylum seekers and refugees Most vulnerable groups are prioritised National, returned and displaced populations were included	Cash transfers Direct food provision -Hygiene Kits	Most vulnerable individuals/families are prioritised
	*Ingreso solidario (IS)*	New transfer	Regular migration status [PEP for Venezuelans]	Limited to regular migrants registered in social programmes registry (SISBEN)	Irregular migration status				
Ecuador	*Bono de Desarrollo Humano* (BDH)	Maintenance	Ecuadorian nationality [since 2019 for BDH]	Exclusion	Migrants and refugees are not eligible	Transfers to NGOs and local governments	Open to migrants, asylum seekers and refugees Most vulnerable groups are prioritised	Cash transfers Direct food provision Hygiene kits	Open to migrants, asylum seekers and refugees
	*Bono de Protección Familiar por Emergencia* (BPFE)	New transfer	Ecuadorian nationality [since 2019 for BDH]	Exclusion	Migrants and refugees are not eligible				
Mexico	*Benito Juárez* (BJ) scholarship for young students^3^	Maintenance	Regular migration status	Limited inclusion [uneven geographical discretionality]	BJ: Uneven criteria for required documentation	Cash transfers Hygiene kits Financing of shelters and lodging	Flexible criteria for NGO financing	National network with comprehensive provision of services in shelters Direct food provision Hygiene kits	Open to anyone in need (nationals and non-nationals)
	*Pensión para el Bienestar de Adultos Mayores* (PBAM)	Early disbursement	Mexican nationality	Exclusion	Migrants and refugees are not eligible				
Peru	*JUNTOS*	Expansion of cash transfer to new recipients	JUNTOS: Specific regular migration status	JUNTOS: Limited to migrants and refugee with residency card	JUNTOS: Asylum seekers, temporary residence permit holders, and irregular migrants are not eligible	Cash transfers Direct food provision Hygiene kits	Open to migrants, asylum seekers and refugees Most vulnerable groups are prioritised	Cash transfers Direct food provision Hygiene kits	Open to anyone in need (nationals and non-nationals) Venezuelan migrants and refugees are prioritised
	Subsidies^2^	New transfers	Peruvian nationality [Valid national ID]	Exclusion	Migrants and refugees are not eligible				
Uruguay	*Asignaciones Familiares Plan de Equidad (AFAM PE)*	Maintenance and increase of existing benefit	Regular migration status [valid national ID issued to asylum and residence permit applicants, *cédula de identidad provisoria*]	Limited to regular migrants	Migrants that do not certify having a residence permit application or asylum seeker application are excluded	Cash transfers Direct food provision Hygiene kits Financing lodging	Open to migrants, asylum seekers and refugees Most vulnerable groups are prioritised Venezuelans and vulnerable migrant groups are prioritised Contingent inclusion of nationals	NGOs receive support from AIs	Open to migrant, asylum seekers and refugees
	*INDA*								
	*Tarjeta Uruguay Social (TUS)*								
	*Canasta de Emergencia Alimentaria (CEA)*	New transfer	Not conditioned to regular migration status	Full inclusion	Administrative challenges for digital registration faced by irregular migrants [These issues were solved through direct provision of food]				

Source: Own elaboration based on the analysis of interviews conducted in the seven country case studies

(1) Chile implemented a wide range of policies and NCSTs based programmes in response to the pandemic. These included the *Bono Covid19* [COVID-19 Benefit] (Law 21,225) and *Ingreso Familiar de Emergencia (IFE)* [Emergency Family Income] (Law 21,230), the former targeting the 60% most vulnerable households and the former targeting households with no formal income, belonging to the 90% most vulnerable. The government also implemented the *Bono a la Clase Media* [Middle-Class Benefit] and the *Subsidio Ingreso Mínimo Garantizado* (Law 21,218) [Minimum Wage Guarantee]. (2) Peru implemented the following NCST programmes: Yo me quedo en casa [I Stay at Home] (Executive order 027-2020, art. 2), Bono Independiente [Self-employed Benefit] (Executive order 027-2020, art. 3), Bono rural [Rural Benefit] (Executive order 042-2020, art. 2), and Bono Familiar Universal [Universal Family Benefit] (Executive order 052-2020, art. 2). (3) As part of the current administration’s non-contributory programs, Mexico also ​​implements the *Programa Pensión para el Bienestar de las Personas con Discapacidad* [Pension Program for the Welfare of People with Disabilities], through which girls, boys, adolescents, and youth aged 0–29 with permanent disabilities, and indigenous population aged 0–64, receive cash transfers in bimonthly basis

In all the countries analysed, there were pre-existing NCST programmes, and in almost all of them—apart from Mexico—new emergency schemes were created (Blofield et al., 2020). However, there is a broad heterogeneity with regards to the degree of inclusion of migrant and refugee populations in both types of programmes. In most countries, access to NCSTs or state aid is conditioned to having an identity card, and in some of them, also being part of social programme registries—which may imply meeting certain income threshold criteria. However, the way in which these programmes have been implemented, especially with regards to compliance with the established requirements, largely determines migrants and refugees’ effective access.

At one end of this social inclusion spectrum are Brazil and Uruguay, which have the highest levels of inclusion. In both countries, the benefits of existing programmes—PBF in Brazil, and *Asignaciones Familiares por Plan de Equidad (AFAM PE)* [Family Allowances through Equity Plan]*, Canasta Instituto Nacional de Alimentación (INDA)* [Food aid from the Food National Institute] and *Tarjeta Uruguay Social (TUS)* [Social Uruguay Card]*,* in Uruguay—were incremented, and in Brazil, there was a significant increase in coverage—1.22 million new families were included (Bartholo et al., 2020; Blofield et al., 2020). Additionally, new transfer programmes, respectively, the *Auxílio Emergencial* [Emergency Allowance] and *Canasta de Emergencia Alimentaria* (CEA) [Food Emergency Aid], were created (Table 2). In both pre-existing and recent transfers, effective access to migrant and refugee populations is guaranteed. In Brazil, the Basic Social Protection System (PSB), which includes health, social assistance, and security for low-income families and/or in conditions of social vulnerability, is universal and guarantees protection regardless of immigration status (MDS, 2016).

In Uruguay, where an application or valid residency permit are a requirement for accessing NCSTs, most migrants have been regularised through various routes. Social programmes (AFAM PE, INDA and TUS) require an identity card (*cédula de identidad*)—a relatively easy document to obtain. However, due to the slowdown in immigration procedures in the context of the pandemic—a phenomenon common to the entire region—some migrants lacked the required documentation to apply for these programmes, as stated by our interviewees from CSOs. However, this obstacle was overcome by in-person delivery of the CEA and facilitating the acquisition of a national ID to the programmes’ beneficiaries, as reported by interviewees from the national government in Montevideo and Rivera. The Uruguayan case exemplifies how bureaucratic-administrative requirements can be subordinate to guaranteeing effective access to rights. Thus, in these two countries, where there is full inclusion and clarity in the legal framework regarding the rights of migrants and refugees and an expanded social protection system, effective access to social protection is verified both in pre-existing and ad hoc programmes created to mitigate the effects of the pandemic.

In the other countries analysed, migrants and refugees face a situation of limited inclusion or outright exclusion. In particular, in Chile, Colombia, Peru, Ecuador and Mexico, the central obstacle for effective access to many social protection programmes is the type or lack of documentation, as well as the lack of awareness regarding some procedures and eligibility requirements on the part of bureaucratic agents as well as the migrant and refugee populations.

In the case of Chile, where the main non-contributory transfer programme has been maintained and new schemes have been created, the main restriction faced by migrants is the nature of the requirements. In order to access the benefits, the person must have an identity card, and in some cases, also be registered in the national social protection registry *Registro Social de Hogares (RSH)* [Household Social Registry]. Thus, the inclusion of migrants and refugees is conditioned by legal status (Interviews with representatives of a migrant organisation and an NGO in Santiago). Although a government interviewee insisted that the criterion for inclusion in pre-existing and new social protection programmes is ‘transversality’, reaching any vulnerable population, in practice, only the regularised migrant population has been able to (partially) benefit from these schemes (Freier & Vera Espinoza, 2021). According to interviewees in Santiago and Arica, migrants with expired identity cards or those with irregular migration status face the greatest difficulties.^6^ A study of the last Chilean National Socioeconomic Characterisation Survey (CASEN) shows that even for those migrants that managed to access government subsidies during the pandemic, the average amount they received was 58.3% below the average amount obtained by the Chilean nationals (Acuña, 2021).

Our interviews suggest that in Colombia, Peru, Ecuador and Mexico, migrants and refugees face the greatest difficulties for effectively accessing NCSTs, not only due to the required documentation but also because in some cases, programmes’ rules are ambiguous, imprecise or these programmes are restricted to the national population. These are cases of outright exclusion, even for those in possession of some form of legal stay. In Colombia, the *Familias en Acción (FA)* [Families in Action] programme is only available to Colombian citizens while *the Ingreso Solidario (IS)* [Solidarity Income] created during the pandemic, is conditioned to having a regular migration status, including the PEP created for the Venezuelan population and being part of the *Sistema de Selección de Beneficiarios para Programas Sociales (SISBEN)*^7^ [System of Identification of Social Programmes Beneficiaries]. In Peru, the pre-existing Programa Nacional de Apoyo Directo a los Más Pobres—JUNTOS [National Programme of Direct Support for the Poorest], is only available to migrants and refugees with a residence permit, which excludes a significant portion of migrants with temporary residence, asylum seekers and irregular migrants (Interview with IOM representative in Tacna). As stated by an interviewee from the Peruvian Ombudsman office, even the president had said in a press conference that support for migrants was to be provided by international cooperation.

Ecuador and Mexico are at the other end of the social inclusion spectrum. In Ecuador, in spite full inclusion of migrant social rights in the legal framework, the pre-existing social protection programme, *Bono de Desarrollo Humano* (BDH) [Human Development Bond], has been limited to Ecuadorian nationals since 2019, and although the new *Bono de Protección Familiar por Emergencia* (BPFE) [Emergency Family Protection Bond] scheme does not explicitly exclude migrants and refugees, in practice this same criterion is applied (Interview with IO staff, Quito). Thus, these populations did not have recourse to social protection either prior to the pandemic, or to the schemes designed to mitigate its impacts.

Unlike the other countries analysed, Mexico had ended its long-term CCT programme, *Progresa/Oportunidades/Prospera* (1997–2018) [Progress/Opportunities/Prosper], before the pandemic and had created three non-contributory schemes for specific populations: the elderly, the young and people with disabilities. The former is limited to Mexican nationals,^8^ while the latter two, formally include the migrant population, but in practice, effective access is contingent upon the discretionality of the political-administrative jurisdiction where the benefit is requested (interviews with NGOs representatives and local authorities in Mexico City and Tapachula). Mexico is the only country analysed where no mitigation measures were implemented to deal with the deleterious social and economic effects of the pandemic.^9^

In recent years, the presence of international organisations such as UNHCR and IOM has been increasing throughout the region, carrying out key actions for the care and protection of migrant and refugee populations, alongside CSOs. As shown in Table 2, in the context of the pandemic, IOs and CSOs had to retool their budgets and action plans to redirect resources, originally allocated to socio-economic integration schemes, towards the expansion of humanitarian assistance programmes.

In all the countries analysed, the initiatives undertaken by the IOs target the entire migrant and refugee populations, while, given budget constraints, their cash transfers schemes are restricted to the most vulnerable. In addition, as stated by our interviewees, in Ecuador, Brazil, Chile, Peru and Uruguay, some funds come already earmarked for the Venezuelan humanitarian response. In Colombia and Mexico, IOs also provide assistance to the returned and displaced national populations. In the context of the pandemic, there was a general relaxation of criteria and requirements for the aid distributed directly or through CSOs in all the countries analysed. For example, the period for receiving cash transfers (normally limited to 3 months) was extended and aid was granted to CSOs that would normally not meet the legal organisational requirements. In addition, in countries such as Mexico, a network of CSOs, created more than three decades ago, extended throughout the national territory, mainly running shelters that provide accommodation, food, medical care and jobs and education-related services.

In sum, during the COVID-19 pandemic, we identified a social inclusion spectrum made up of three groups. At one end of the spectrum, the first group, composed by Brazil and Uruguay, is characterised by the *guarantee and effective access to social rights of migrants and refugees*, where often the bureaucratic-administrative structure is subordinated to the effective exercise of rights. The second group is composed by Chile and Colombia, where *regular migration status conditions access to and exercise of certain social rights*, while at the other end of the spectrum, in the third group, composed by Peru, Ecuador and Mexico, *migrants and refugees are generally excluded from social protection schemes*, often due to the lack of clarity in programmes’ rules of implementation (specially at the local level) and the exclusion of migrants from broader categories of vulnerability*.*

Although the actions of IOs and CSOs display some commonalities in the countries studied, providing an expeditious, relatively flexible response adjusted to the humanitarian needs generated by the pandemic, their role, *vis-à-vis* the actions of the state, differs between countries, as we discuss below.

## Towards a typology of models of social protection in the context of COVID-19

The analysis of NCST programmes and initiatives in the seven countries, before and after the pandemic, shows an increase of emergency assistance that is consistent with the increasing needs, and exacerbated vulnerabilities produced by the sanitary-economic crisis. However, who provides the assistance, what type of assistance is provided and the extent to which migrant and refugee populations are included in these initiatives, varies across countries.

It is worth noting that the type of assistance—whether emerging from state-led responses or those funded and distributed by IOs and CSOs, or from the articulation of both—does not radically differ from the pre-pandemic situation. There is also little variation in the logic of inclusion/exclusion that existed before the pandemic, in terms of who can access social assistance and under what terms.^10^ Nonetheless, the examination of migrants’ inclusion in NCSTs during the pandemic provides a window for the identification of different models of social protection in the region.

In Table 3 we propose a typology of the models of social protection during the pandemic in the seven case studies, in relation to three key aspects: (1) the assemblage of actors providing social protection during the COVID-19 pandemic, (2) the modes of protection and, (3) the migrants’ subjectifications that emerge from these configurations.

**Table 3:** A typology of models of social protection in the context of COVID-19 pandemic

Country	Assemblages of actors providing social protection to migrants and refugees during pandemic	Modes of protection during the pandemic	Migrant subjectification during pandemic
Brazil	State-led with complementary role of IOs and Civil Society	Rights based—adapted in the context of emergency	Subject of rights
Chile	State led and complementary role of IOs and Civil Society	Rights based in principle, contingent in practice	Humanitarian subjects
Colombia	IOs and Civil Society led and complementary role of the state	Contingent	Humanitarian subjects
Ecuador	Absent state, response led by Civil Society and IOs	Marginal contingent	Humanitarian subjects
Peru	Absent state, response led by Civil Society and IOs	Contingent	Humanitarian subjects
Mexico	Absent state, response led by IOs and Civil Society	Contingent	Humanitarian subjects
Uruguay	State led and complementary role of IOs and Civil Society	Rights based—adapted in the context of emergency	Subject of rights

Source: Own elaboration based on the evidence presented in Tables 1 and 2

### Assemblage of actors

This typology considers the articulation of different actors in the provision of social protection, primarily through NCSTs, during the COVID-19 pandemic. We identified three assemblages of actors:

1. State-led with complementary role of IOs and Civil Society
2. IOs-led and complementary role of the state and Civil Society
3. Absent state (central), response led by Civil Society and IOs

The articulation and varying roles of the different actors during the pandemic bring to the fore the modes of protection and the systems of governance that may emerge after the pandemic, as we discuss below. While in Brazil, Chile and Uruguay, IOs and CSOs play a complementary role to government actions; in other cases, their role is rather supplementary. In Colombia, they lead social protection efforts and complement the actions of the national government, while in Ecuador, Peru and Mexico they lead protection efforts and make up for the absence of the state.

In Brazil and Uruguay, which already had a structure of social protection prior to the pandemic, central governments have effectively included migrants, regardless of migration status, into the social protection mitigation measures (interviews with CSOs in São Paulo and Boa Vista, Brazil, and national government representatives in Uruguay). In turn, IOs and civil society have had a complementary role developing an emergency response aimed to address the basic needs of the vulnerable population. Similarly, Chile developed an emergency response led by the central government that, although in principle does not exclude the migrant population, in practice the eligibility criteria limits access to migrants and refugees who have a regularised status and meet certain criteria (see Table 2). IOs and CSOs have a complementary but crucial social protection role towards all migrants and refugees in the country, and in some cases even including national residents, as evidenced by our interviews with IOs and faith-based NGO representatives. A key aspect to these dynamics is the partnerships that UNHCR and IOM have established with some Chilean municipalities. For instance, as stated by our IOs interviewees, by July 2020, UNHCR had worked with the Municipality of Santiago to provide food and shelter and with the Municipality of Estación Central, to provide 200 food baskets, 50 hygiene kits and 40 kits of diapers; while IOM has partnered with some private local businesses to directly deliver food baskets to migrants. The municipalities of Santiago and Arica have also been working closely with NGOs and migrant-led organisations to cover the basic needs of the local migrant population and of those waiting to return to their countries of origin (see Vera Espinoza et al., 2020).

A different type of assemblage is observed in Colombia, where the emergency response (inclusive of migrants) has been led by both the IOs and CSOs, with a complementary role of the state. The central government claims that they have coordinated international cooperation efforts (Presidencia de la República de Colombia, 2020), but in practice they have limited their action to providing cash-transfers to a fraction of the resident migrant population (less than 20 thousand Venezuelans) and food baskets to vulnerable families (Interview with national government representative).

In Peru, we identified an absent state in relation to the provision of social protection. The emergency response has been led by IOs, mainly UNHCR and IOM, with a complementary role by CSOs. As in the case of Chile, in Peru we also found that local governments (Tumbes and Tacna) have partnered with IOM and UNHCR. As part of this response, IOs have expanded their vulnerability criteria beyond the migrant population, in an attempt to avoid fuelling the already high levels of xenophobia (Interview with IOM representative in Tacna).

Similarly, in Ecuador, the lack of inclusion of migrant and refugee populations in social protection schemes translates in an absent state to respond to the increasing social needs provoked by the pandemic, where IOs, especially UNHCR and IOM, and CSOs have led emergency assistance efforts. Also, some humanitarian actions such as distributing food and cleaning kits, were carried out by the Pichincha and Carchi local governments and other southern border towns during the most critical months of the pandemic (Interviews with local government officials and IO representative).

Mexico is another country with an absent state in relation to the provision of social protection. This absence should be understood both in relation to the sanitary crisis and to changes in migration policy enacted before the pandemic. The government of Lopez Obrador (2018—current) has embraced enhanced securitisation and deterrent practices as core elements of migration policy,^11^ increased institutional instability (through the externalisation of US border controls within the Migration Protection Protocols Programme) and ratcheted up migrant detention. In addition, the pandemic reached Mexico in a context of weak or non-existent social protection programmes for migrant and refugee populations, with NGOs leading the emergency response.^12^ IOM and UNHCR have also had an increasingly important role, not only providing NCSTs (such as shelter and cash transfers), but also supporting the range and quality of action of NGOs through capacity building and infrastructure.

In sum, the assemblage of actors varies across countries, and even in countries where social protection systems fully include the migrant population, such as Brazil, the different modes of articulation do not necessarily translate into effective inter-sectorial coordination, as emphasised by our interviewees. These different assemblages of actors may relate, to some extent, to the lack of reception structures in some countries of the region, even in those with progressive legal frameworks, such as Ecuador. This is consistent with changing migration dynamics across the region, with countries transitioning from mainly sending countries (such as Colombia) or transit countries (such as Peru and Mexico) to key intra-regional destination countries. Other countries in the region with mixed migration profiles, such as Brazil, Uruguay, Chile and Ecuador, have variable levels of reception structures, evidenced by the different articulation of actors in the provision of social assistance. This in turn, shapes the outcomes in the modes of protection, as we discuss below.

The key role of civil society organisations in providing social protection for migrant and refugee populations in Latin America is long dated, and has been especially forceful since the return to democracy (Avritzer, 2007). What is relevant in the current context, is how many of these NGOs and faith-based organisations, albeit their limited resources, have quickly addressed some of the shortcomings in social protection that emerge from the corseted structures of both central governments and IOs, while still closely working with them (see Nair et al., 2021). As the interviews in the seven countries revealed, these organisations have demonstrated a speed for response and organisation that far exceeds that of governments, through the retooling of their budgets and action plans to effectively respond to the pandemic. Equally relevant has been the role of migrant-led organisations at both the local and national levels: interviewees from Ecuador, Chile, Colombia and Peru, highlighted how they diversified their range of action to include the provision of food baskets, *ollas comunes,* advocacy and information campaigns during the pandemic.

Another key point is the relevance of the local level. Municipal governments have been closely working with all the other actors identified in the provision of social protection as they are, alongside civil society, a focal point of contact for migrant and refugee populations (Bengochea et al., 2021; Vera et al., 2020). Although in Table 3 we mainly refer to central governments, as the main designers of social protection programmes, in countries such as Ecuador, Chile and Colombia, municipal governments have had a key role in the distribution of the emergency assistance on the ground.

Finally, our interviewees emphasised that the assistance provided by IOs and CSOs in the countries of the region is conditioned by funding and by the temporal limitations that involve an ‘emergency response’, raising questions on the effectiveness of these efforts in relation to migrants’ long-term integration. At the same time, these actions, while needed, fail to challenge the exclusionary status quo of current states’ practices in the region, which condition the ‘deservedness’ of protection to a regular status (Ehrkamp & Nagel, 2014).

### Modes of protection

We identified four modes of social protection, according to the different assemblages of actors in the seven country case studies, in the context of the COVID-19 pandemic (Table 3).

#### Rights based: adapted in the context of emergency

Social protection for migrant and refugee populations is integrated into national social protection systems. New programmes emerge to respond to the sanitary-economic crisis, which are complementary to the state’s existing safety-net programmes. Access to these programmes is not determined by immigration status, but rather by levels of vulnerability (mainly through the flexibilization of some eligibility criteria). This is exemplified by the cases of Brazil and Uruguay.

#### Rights based in principle, contingent in practice

Social protection for migrant and refugee populations is integrated into national social protection systems, with new emergency programmes complementing the existing safety-net programmes. Access to these programmes is determined by levels of vulnerability, subject to migrant regularisation and specific proof of ID, among other specific requirements. Chile fits this profile.

#### Contingent

In this mode of social protection, emergency programmes, that selectively include migrant and refugee populations, emerge outside of the national social protection system. The emergency response is short-term, targeted and conditioned to levels of vulnerability among these populations. This is exemplified by the cases of Colombia, Peru and Mexico.

#### Marginal: contingent

Migrant populations are not included in national social protection systems and are actively excluded from emergency responses led by central governments. Migrant and refugee populations are included in programmes developed by IOs and CSOs, based on broad vulnerability criteria. Ecuador fits this profile.

### Migrant subjectification

This classification alludes to emerging subjectifications of migrants, as a result of both the assemblages of actors who provide assistance and the modes of protection identified.

We identified two broad, and by no means, exhaustive constructions:

1. Migrants are considered as *subjects of rights*, regardless of migration status.
2. Migrants are rendered as *humanitarian subjects*, as a result of assistentialist short-term models of protection, as their access is both determined and constrained by the ‘emergency’. Migrants’ access to social protection seems to be understood in line with humanitarian ideas of ‘compassion’ and the externalisation of assistance, as well as determined by residency and/or regular migration status.

In Brazil and Uruguay migrants emerge as subjects of rights, as they are included—without restrictions—in the provision of social protection. This assertion, however, does not provide a full account of migrant and refugee populations’ effective access to a wider range of social, economic, and cultural rights in these countries. Yet, the existence of a state that leads the protection response, does not necessarily guarantee migrants’ inclusion as subjects of rights. As evidenced by the Chilean case, migrants’ access to social assistance has been contingent to the emergency and limited to regular migration status, among other conditionalities. In this context, migrants and refugees are tacitly understood as *humanitarian subjects*.

In countries where there is total or partial absence of the state regarding the protection of migrants and refugees, such as Peru, Ecuador Colombia and Mexico, migrants have been rendered as *humanitarian subjects*, as recipients of humanitarian help from IOs and NGOs, with limited access to long term sustainable solutions. In this regard, the Ecuadorian case is emblematic: although the constitution guarantees equality of rights for nationals and non-nationals, the government closed all avenues for social protection to the migrant community before and during the pandemic.

In the Peruvian case, the construction of migrants as *humanitarian subjects*, in a context of state absence, begins at the design stage of aid initiatives by the IOs, where compliance with the vulnerability requirements defines the target population. As pointed out by our interviewees in Peru, the state uses its sovereign powers to place migrants in a *legal limbo*, so that migrants have recognised rights that are not effectively available in practice.

The five countries where the emerging, and in some cases, continuous construction of migrants as *humanitarian subjects*, evidence a lack of inclusion of migrant and refugee populations in social protection, with consequences beyond this realm alone. On the one hand, the overreliance on regular status as conditionality to access rights and protection reinforces the logic of restrictions seen across the region (Domenech, 2011). These selective inclusion practices tend to reinforce inequalities, and migrants’ experiences of precarity and vulnerability, especially in times of crisis. On the other hand, the construction of *humanitarian subjects* establishes contingent policies as the norm, which are tightly linked to the exceptional treatment given, in particular, to recent mixed migration flows across the region. This is leading to states’ disengagement from their responsibility of social protection to subjects of human rights, relying on non-state actors, externalising, or avoiding the development of policies and practices of inclusion.

## Conclusions

Our analysis shows that the responses to the pandemic developed in the seven countries studied, reflect a continuity, and further normalisation, of existing practices along a spectrum of inclusion/exclusion that preceded the COVID-19 outbreak, but with new configurations with regards to the assemblages of actors providing social protection. Our proposed typology of models of social protection in the context of the pandemic, varies according to the actors involved, the modes of protection and the conception of migrants as *humanitarian subjects* or *subjects of rights.*

The findings suggest broad heterogeneity and complexity with regards to different degrees of inclusion for migrant and refugee populations, particularly in pre-existing and new NCST programmes. On the one hand, Brazil and Uruguay clearly stand out for having fully inclusive and clear legal frameworks, the enhancement of pre-existing programmes, and the creation of new ones that fully include displaced populations regardless of immigration status. On the other hand, the evidence collected in the other five countries—Chile, Colombia, Ecuador, Mexico and Peru—allows us to dwell on the challenges faced in Latin America concerning the tensions and contradictions between somehow advanced legal frameworks, however ambiguous, and policy implementation. It also allows for a better understanding of the articulation of actors in the provision of social protection, as well as the strategies and practices they deploy. We observe a common pattern in which actors prioritise the provision of basic needs, while sacrificing existing plans and programmes, which may negatively impact migrants’ medium- and long-term processes of integration.

The evidence presented here contributes to advancing three key discussions. First, it brings to the fore how global discourses on humanitarian crises and the attendant regional articulation of a “crisis within the crisis” have contributed to the state’s disengagement and the deterioration of effective policies of social protection for migrants and refugees in Latin America. That is to say that the socioeconomic mitigation measures put in place to deal with the sanitary-economic crisis are based on and further affirm notions of protection that are contingent and crisis-driven, with temporal limitations that often selectively exclude migrants based on legal status.

Second, we mobilise understandings about the key role of social rights as a basic condition for the effective integration of migrant and refugee populations within a framework of rights-based citizenship. Third, we have shown the complexities of the nature of policies and practices of migration governance in Latin America. The new legal categories and *ad-hoc* measures that emerged across the region in response to the displacement of Venezuelans and Central Americans, among other international mobilities, have contributed to either produce migrant irregularity or to reinforce practices of exclusion/inclusion that impact on migrants’ effective access to social and economic rights. We discuss all these aspects in relation to migrant subjectification as either *subjects of rights* or *humanitarian subjects*. Our intention with this categorisation is by no means to produce yet another binary understanding of the migrant subject. Rather, our analysis sheds light on the diverse current assemblages of actors and the social inclusion/exclusion spectrum operating in the region, and how they are shaping migrants and refugees’ lives in Latin America, through an enhanced understanding of their rights and effective access to social protection during the pandemic. In this way, we contribute to expanding a growing body of literature on social protection and migration governance in Latin America and on migrants’ and refugees’ integration, while paving the way to keep exploring the differentiated impacts of the COVID-19 pandemic on (im)mobility across the region.

## Data Availability

The interview material generated for the current study is not publicly available due to confidentiality obligations towards the study participants. No names have been used in the paper to respect these obligations. The data presented in this paper is part of a larger study, which other outputs are still under development.

## Appendix

See Table 4.

**Table 4:** List of interviews by country and city

Country	City	Type of organisation
Brazil	São Paulo Boa Vista	3 International Organisations
		1 representative from federal government
		1 representative from local government
		6 Civil Society Organisations
Chile	Santiago Arica	2 International Organisations
		2 representatives from national government
		1 representative from local government
		5 Civil Society Organisation
Colombia	Bogotá Cúcuta	2 International Organisations
		1 representative from national government
		6 Civil Society Organisations
Ecuador	Quito Tulcán	2 International Organisations
		2 representatives from local government
		2 representatives from national government
		6 Civil Society Organisations
Mexico	Mexico City Tapachula Tijuana	3 International Organisations
		2 representatives from local government
		5 representatives from national government
		10 Civil Society Organisations
Peru	Lima Tacna Tumbes	3 International Organisations
		1 representative from local government
		4 representatives from national government
		6 Civil Society Organisations
Uruguay	Montevideo Rivera	2 International Organisations
		2 representatives from local government
		3 representatives from national government
		3 Civil Society Organisations

Sources: Own elaboration based on interviews

## Abbreviations

AFAM: *Asignaciones familiares*
*AFAM PE*: *Asignaciones familiares plan de equidad*
AE: *Auxílio emergencial*
PSB: Basic social protection system
BJ: *Benito juárez*
PBF: *Bolsa família*
BDH: *Bono de desarrollo humano*
BPFE: *Bono de protección familiar por emergencia*
CEA: *Canasta de emergencia alimentaria*
CSOs: Civil society organisations
CCT: *Conditional cash transfer*
FA: *Familias en acción*
*IS*: *Ingreso solidario*
IOM: International Organisation for Migration
IOs: International organisations
NCST: Non-contributory social transfer
NGOs: Non-governmental organisations
PBAM: *Pensión para el bienestar de adultos mayores*
PEP: *Permiso especial de permanencia*
*SUF*: *Subsidio único familiar*
*TUS*: *Tarjeta uruguay social*
*TUS-PE*: *Tarjeta uruguay social plan de equidad*
UNHCR: United nations high commissioner for refugees

## Footnotes

1 Through the paper we use the term migrant and refugee populations to refer to all people crossing international borders across the region, without making a distinction by migration status. To reduce wording, we may use the word migrants, without excluding refugees or asylum seekers.

2 The interviews were conducted by a team of 18 people, including all authors and six collaborators.

3 Given the specific border dynamics in Peru and Mexico, interviews in these countries included two border cities, increasing the overall number of interviews in these countries.

4 The research received approval by the Ethics Committees of Queen Mary University of London (QMERC2020/27) and the Federal University of Minas Gerais (CAAE: 34657020.1.0000.5149).

5 Although outside the temporal scope of this paper, in February 2021, Colombia took an unprecedented measure for the legal social and economic integration of Venezuelans by announcing the Estatuto Temporal de Protección al Migrante Venezolano (ETPMV), a ten-year Temporary Protection Status scheme.

6 Although outside the temporal scope of this paper, there are two relevant aspects to highlight here: (1) Article 16 of the new migration law promulgated in April 2021 (Law 21325) establishes a 2-year residence requirement to access benefits and NCSTs that involve resources from the Chilean state. This law will enter into force once its regulations are published. (2) In August 2021, the government announced that non-nationals without RUN (national ID number), specifically those with Chilean children and those who have a visa pending approval (requested until July 1, 2021), would be able to apply to the Ingreso Familiar de Emergencia (IFE) [Emergency Family Income] making a request online. However, soon after implementation complaints have emerged in social media about the functionality of the platform.

7 The System of Identification of Social Programmes Beneficiaries (SISBEN) established a household vulnerability index used to identify the beneficiaries of social assistance programmes.

8 An agreement is being formalised in some cities to include a limited number of young refugees in the programme (Interview with a COMAR Commissioner, June 2020).

9 In Mexico City, migrants, asylum seekers and refugees are eligible to access the *Programa Ciudad Hospitalaria* [Hospitable City Programme] which offers temporary unemployment compensation, access to jobs and training, health services and temporary housing. During the first months of the pandemic this programme was suspended and later began to gradually reopen. For example, in the case of access to health, the programme links migrants with specific clinics in their host city, sincemany clinics do not recognise their documents or accept them without documents. Thus, migrants’ access to health is not guaranteed, but depends on the discretionality of public servants and health worker professionals.

10 While many of the requirements to access social protection did not change during the pandemic, some countries introduced different ways to identify the programmes’ target population. For instance, Chile developed a new Socioeconomic Emergency Indicator (ISE), to measure the short-term impact of the pandemic on households’ socio-economic conditions, although this instrument is no longer in use (Berner & Van Hemelryck, 2021).

11 With the arrival of the migrant caravans—between late 2018 and early 2019, US President Donald Trump threatened Mexico with trade tariffs unless the Mexican government stopped the flow of Central Americans on route to the United States. Mexico responded by enacting three key migration policy changes: (1) the intensification of the Migrant Protection Protocols; (2) the militarisation of migration control, with the creation of the National Guard and; (3) an institutional reorganisation of migration governance that passed over the responsibility of migration controls from the Ministry of the Interior to the Ministry of Foreign Relations (Gandini 2020).

12 Prior to the pandemic, Seguro Popular, a programme that provided temporary health coverage to those without insurance, including migrant and refugee populations, was abolished. As a result, 15 million people lost access to health services between 2018 and 2020 (CONEVAL 2021). The *Instituto de Salud para el Bienestar (INSABI)* [Institute of Health for Welfare] was created under the current administration. However, to date it does not have clear implementation rules. The shortcomings of both programmes, before and during the pandemic, mean that the provision of public health tends to be case-by-case and challenged on a daily basis at the local level, affecting migrants and refugees, who are commonly left with no coverage. During the pandemic, protocols regarding medical and mental health services for immigrant populations were published (Bautista-González et al., 2021), but there is not enough evidence on how they were implemented.

## Declarations

### Data collection

The interview team (18 people) was formed by all the authors of this paper and six collaborators (17 women and 1 man). All these collaborators are included in the acknowledgment section. All interviewers have experience in research on migration—between 2 and 10 years, and they are based on institutions in Latin America and Europe. In some cases, interviewer and interviewee knew each other from interviews for previous studies, but it was not always the case. All interviewees were contacted by email and provided informed consent ahead of the interview.
